# The moderated mediating effect of gender in the relationship between unemployment, depression, and suicide during the COVID-19 pandemic: An examination based on big data

**DOI:** 10.1192/j.eurpsy.2022.369

**Published:** 2022-09-01

**Authors:** Y. Noh, H.A. Kim, S.B. Lee

**Affiliations:** JeonBuk National University, Social Welfare(bk21 Four), Jeonju-si, Republic of Korea

**Keywords:** Covid-19, Depression, unemployment, Suicide

## Abstract

**Introduction:**

The COVID-19 pandemic, and the consequent recession, have caused a decline in the job market, with the resultant job insecurity increasing the risk of depression. While this affected all genders, suicidal thoughts were observed to be more common among women than men, suggesting that the impact of unemployment on depression varies by gender, with gender differences affecting the outcome of depression.

**Objectives:**

This study aims to verify the moderating effect of gender on the structural relationship between unemployment, depression, and suicide during the COVID-19 pandemic by using online search trend data.

**Methods:**

The study utilized the search trend data from Naver’s Data Lab service, by analyzing the searches of men and women under 65, between March, 2020 and September 12, 2021. The search terms were “unemployment,” “depression,” and “suicide.” The analysis examined 1121 searches using the Model 7 research model through the SPSS Process Macro to verify the moderating effect of gender on the mediating pathways for unemployment, depression, and suicide.

**Results:**

We observed that searches for “unemployment” significantly increased with searches for “depression” (B=1.860, p<.001) and “suicide” (B=.860, p<.001). The analysis further revealed that the correlation between the increase in searches relating to depression and unemployment was seen more in women than men. This resulted in an accompanying increase in the volume of searches for suicide (B=2.341, p<.001).

**Conclusions:**

The job insecurity caused by the COVID-19 pandemic led to varying degrees of depression according to gender. Thus, social security measures related to unemployment, depression, and suicide interventions require a gender-specific approach.

**Disclosure:**

No significant relationships.

